# Mitral Valve Repair vs. Replacement: Hemodynamics in Valve Surgery

**DOI:** 10.31083/RCM49299

**Published:** 2026-06-28

**Authors:** Mingrui Zou, Kang Huang, Xiaoning Wang, Shuaishuai Zhao, Bo Wu, Xin Hong, Xueliang Zhou

**Affiliations:** ^1^Department of Cardiac Surgery, The First Affiliated Hospital, Jiangxi Medical College, Nanchang University, 330209 Nanchang, Jiangxi, China; ^2^Cardiovascular Surgery Department, Binzhou Medical University Hospital, 256601 Binzhou, Shandong, China

**Keywords:** mitral valve regurgitation, vortex kinetic energy, cardiac valve surgery, hemodynamics

## Abstract

Mitral regurgitation (MR) is a common valvular heart disease. In the surgical management of MR, mitral valve repair offers significant advantages over replacement by allowing for the optimal preservation of the native valve structure and function. Studies indicate that, compared to valve replacement, repair procedures are superior in restoring physiological left ventricular flow patterns—specifically by optimizing the distribution of kinetic energy, increasing the proportion of direct flow, maintaining normal vortex structures, and effectively reducing energy loss. Replacement with either mechanical or bioprosthetic valves, while effectively correcting regurgitation, substantially alters the left ventricle fluiddynamic environment, leading to increased energy loss and an elevated thrombotic risk. This review discusses the impact of mitral valve repair versus replacement on intra-left ventricular hemodynamics. It further proposes that re-establishing organized left ventricular flow patterns, rather than merely achieving imaging-based improvements, represents a new cornerstone for achieving favorable long-term patient outcomes.

## 1. Introduction

With the ongoing aging of the population, valvular heart disease has become a major cardiovascular condition severely impacting public health. Whether etiology is rheumatic or degenerative, the integrity of the mitral apparatus plays an important role in maintaining left ventricular function. The relationship between mitral valve structure and left ventricular dynamic performance is most important; any disruption of its physiological structure may cause long-term damage to left ventricular function [1,2].

Mitral regurgitation (MR) is the most common type of valvular heart disease in adults. Its prevalence in the Chinese population over 60 years of age ranges from 6.4% to 9.3%. The annual mortality rate for symptomatic MR patients not undergoing surgery is 5%, while for those developing heart failure, the 5-year mortality rate reaches 60%. In 2023, among approximately 2.66 million hospitalized patients with valvular heart disease nationwide, mitral valve disease was the most frequent (50%), with a total of 54,000 mitral valve surgeries performed. Replacement accounted for 65.6%, while valve repair constituted 34.4%. Compared to 32.4% in 2020, 29.4% in 2021, and 25.5% in 2022, the proportion of repair surgeries has shown a steady downward trend, reflecting the refined development of mitral valve repair techniques [3].

The principle of “repair over replacement” advocated by renowned cardiac valve surgeon Professor Meng Xu is based on the ability of mitral valve repair to maximally reconstruct the normal tissue physiology of the valve. This effectively eliminates anticoagulation-related complications while significantly reducing the risk of adverse events such as infective endocarditis and thromboembolism [4]. This review aims to systematically compare the impact of mitral valve repair versus replacement on left ventricular hemodynamics from a fluid dynamics perspective and discuss its clinical significance.

## 2. Analysis of the Pathological Mechanism of Mitral Regurgitation

### 2.1 Mitral Valve Structure and Physiological Function

**Fig. 1.**
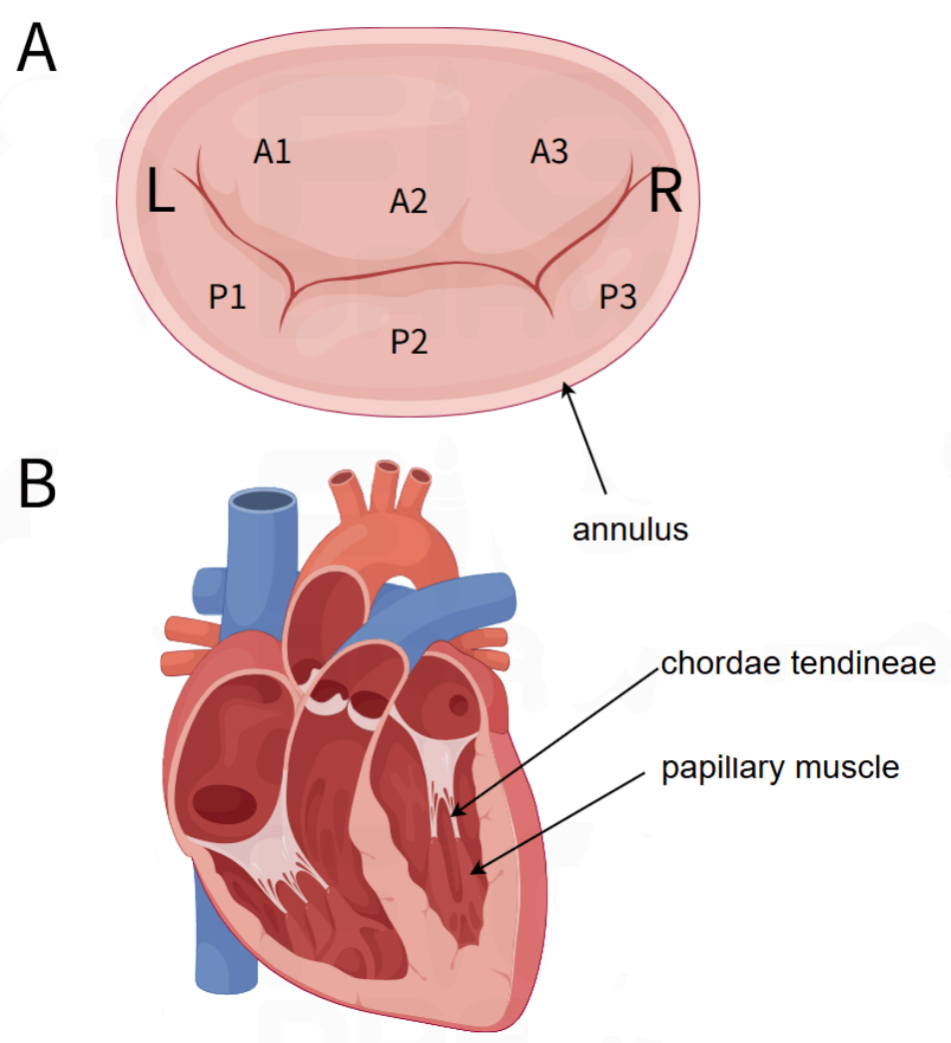
**Diagram of the mitral valve anatomy**. (A) Dark area: The mitral valve leaflets and their divisions. The anterior leaflet A1 is close to the anterolateral commissure (L); A2: The middle part of the leaflet; A3: Close to the posteromedial commissure (R); The posterior leaflet corresponds to the divisions P1, P2, and P3 of the anterior leaflet. Light area: The valve annulus (B) An anatomical diagram of a normal mitral valve (chordae tendineae and papillary muscles) observed from the left ventricular anterior-lateral incision.

The mitral valve (MV) is a precisely structured functional complex, composed of four synergistic components: leaflets, annulus, chordae tendineae, and papillary muscles. Through the precise opening and closing, it ensures unidirectional, efficient blood flow from the left atrium to the left ventricle, forming the basis for maintaining effective cardiac pumping and systemic organ oxygenation. Any structural abnormality can lead to hemodynamic disturbances and a series of cardiovascular diseases [5,6] (Fig. 1).

### 2.2 Fundamental Mechanism and Long-Term Impact of Insufficient Coaptation Area Leading to Regurgitation

The etiological mechanisms of mitral regurgitation are varied and include mitral leaflet prolapse, secondary leaflet tethering, and chordal rupture. The underlying pathology of regurgitation lies in the insufficient physiological coaptation area between the anterior and posterior leaflets. These result in high-velocity systolic blood flow passing through the non-coapted gap and regurgitating into the left atrium [7,8,9].

Mitral regurgitation reduces left ventricular pumping efficiency because part of the stroke volume does not enter the aorta but returns to the left atrium. This process activates metabolic feedback mechanisms, enhancing left ventricular function, which further increases ventricular load, especially during exercise or stress. The most significant pathological consequence of severe regurgitation is left atrial dilation-the atrium must accommodate the additional regurgitant volume and withstand ventricular systolic pressure. Significant atrial dilation indicates that mitral valve prolapse requires clinical intervention [10,11,12].

Mitral valve repair is currently the most commonly used surgical treatment. It reconstructs the patient’s native valve structure and function by resecting redundant leaflet tissue and suturing autologous tissue into an ideal geometric shape. Current clinical evaluation primarily focuses on achieving an ideal valve orifice area to reduce the severity of the regurgitation and lower transvalvular pressure gradients. However, the potential impact of repair on left intraventricular flow patterns is seldom systematically assessed and monitored [13,14].

## 3. The Invisible Skeleton Within the Heart: How Vortex Structures Govern Left Ventricular Pumping Efficiency

Intracardiac blood flow is not a simple linear path; its flow pattern is characterized by multi-scale, rotational vortices. These vortices dynamically change in morphology and scale, ranging from local rotations confined to small regions to large-scale macroscopic structures dominating the flow field, namely, vortices. Each vortex is a dynamic entity with defined physical properties, possessing identifiable volume, relatively fixed spatial position, and specific rotational direction [15,16].

The left ventricle is far more than a passive receptacle or a simple mechanical pump. The realization of its efficient pumping function highly depends on the natural formation of a “large-scale” vortex during diastolic blood inflow. Although this vortex is not directly visible in traditional anatomy, it is the hydrodynamic core that maintains efficient cardiac work. It can guide blood flow smoothly towards the outflow tract, significantly reducing energy loss, suppressing abnormal turbulence, and effectively preventing blood stasis in the apex. Vortices constitute an “invisible functional skeleton” within the heart, having a strong influence on overall dynamic efficiency [15,17,18].

When this “vortex state” is disrupted by surgery or disease, even if postoperative imaging shows an ideal valve orifice area, near-elimination of regurgitation, and reduced pressure gradients—all of which are “perfect” indicators—the overall hemodynamics within the left ventricle may still be in a disordered or inefficient state. This also explains why some patients show significant imaging improvement postoperatively, yet due to abnormalities in fluid dynamics, cardiac functional recovery falls far short of expectations.

## 4. Comparison of Hemodynamic Parameter Changes Post Mitral Valve Repair and Replacement

Hemodynamic alterations are closely related to the development of cardiovascular diseases. Therefore, quantifying relevant hemodynamic parameters has significant reference value [19]. Current quantitative assessment of intracardiac flow in healthy populations and cardiac disease patients using four-dimensional flow cardiac magnetic resonance imaging (4D Flow CMR, see **Supplementary Materials** for details) provides new insights into complex intracardiac hemodynamics. Quantitative hemodynamic parameters include: Kinetic Energy (KE), flow components, vortices and vorticity, viscous energy loss, and hemodynamic forces [20,21,22,23].

### 4.1 Kinetic Energy (KE)

KE reflects the energy possessed by moving blood and is an important indicator for assessing cardiac work efficiency. In the left ventricle, KE can be used to assess flow efficiency; abnormal increases or decreases are associated with cardiac dysfunction [24].

Mitral valve repair preserves the patient’s own mitral apparatus (leaflets, chordae, papillary muscles), maintaining normal left ventricular mechanical coupling. After correcting regurgitation, the abnormally high-kinetic-energy turbulence caused by regurgitation disappears. Simultaneously, as ventricular geometry and contraction patterns are preserved, the heart can more effectively convert blood kinetic energy into forward ejection force. Therefore, overall KE decreases (due to reduced inefficient regurgitation), and the kinetic energy distribution of diastolic flow may more closely resemble the healthy state. In contrast, after valve replacement (especially with mechanical valves), leaflet opening and closing generate high-velocity, localized jets. These jet regions may produce locally increased KE. Furthermore, if subvalvular structures are excised during surgery, normal mechanical conduction within the left ventricle is disrupted, potentially reducing overall ventricular contraction efficiency and affecting the global kinetic energy pattern. Abnormal late diastolic KE may persist postoperatively, which is more common after replacement [17,25].

### 4.2 Flow Components

Left ventricular flow is divided into four components: ① Direct flow: blood entering during diastole and ejected during systole (efficient component); ② Retained flow: blood entering but not ejected; ③ Delayed flow: blood already in the ventricle, ejected during systole; ④ Residual flow: blood persisting for at least two cardiac cycles, not participating in inflow/outflow. A higher proportion of direct flow indicates better cardiac function [25,26].

Mitral valve repair, while correcting regurgitation, optimally restores the physiological flow path within the ventricle. Therefore, the proportion of direct flow (the most efficient component) is expected to increase significantly, while residual and retained flows caused by regurgitation and stasis are greatly reduced. Improvement in flow components is an important marker of successful repair. Similarly, replacement also eliminates regurgitation, thereby increasing direct flow and reducing residual and retained flows. However, prosthetic valves (especially mechanical valves) themselves introduce certain flow resistance and non-physiological flow pathways, potentially generating minute, new retention areas or small vortices. Consequently, the conversion efficiency of flow components may not be as perfect as with a repair [27].

### 4.3 Vortices and Vorticity

① Vortices: rotating structures formed by blood flow; physiological vortices aid efficient filling and ejection. ② Vorticity: quantifies local flow rotation intensity; a sensitive indicator of diastolic function. Abnormal vortices or reduced vorticity indicate ventricular dysfunction [28].

Mitral valve repair preserves the natural geometric morphology of the mitral orifice and the guiding effect of papillary muscles-chordae on flow. This allows diastolic flow to form more regular, physiologically-approximate vortices within the left ventricle. This efficient vortex aids blood mixing and orderly movement towards the outflow tract, thereby improving filling and ejection efficiency. Prosthetic valves (especially bileaflet mechanical valves) generate characteristic, multiple high-velocity jets. These jets disrupt the left ventricle’s inherent, large-scale physiological vortex ring structure, replacing it with multiple smaller, jet-driven vortices. Therefore, vortex morphology, location, and intensity are altered, and their physiological efficiency is typically lower than the natural vortices formed post-repair [29,30].

### 4.4 Viscous Energy Loss

Viscous Energy loss is converted to heat due to friction between the blood and the ventricular wall. This reflects flow efficiency; abnormally high values indicate uneconomical energy utilization [31].

Valve repair, by eliminating regurgitation-a major source of energy waste-and restoring smooth flow pathways, can maximally reduce viscous energy loss caused by turbulence and friction. Valve replacement, while eliminating the substantial energy loss from regurgitation, introduces new flow resistance and local turbulence due to the prosthetic valve itself, particularly its sewing ring, stent, and small orifice, leading to viscous energy loss. Therefore, the overall level of postoperative energy loss may still be higher than that of the physiological repair [30,32].

### 4.5 Hemodynamic Forces

Hemodynamic forces reflect force exchange between intracardiac blood volume and the ventricular wall, a potential marker of early cardiac dysfunction [33].

Mitral valve repair, by preserving the intact structure and function of the left ventricle, is most conducive to reducing ventricular load and restoring a normal mechanical environment. Therefore, the global forces exchanged between intraventricular blood and the wall (HDF) can more rapidly and completely return to normal patterns. This aids in reversing adverse ventricular remodeling and improving long-term prognosis. Mitral valve replacement (especially when combined with excision of subvalvular structures) alters the left ventricular mechanical environment, potentially causing local wall motion abnormalities. This may lead to incomplete recovery of HDF. Abnormal HDF patterns may persist, resulting in a relatively higher risk of future ventricular remodeling [34,35,36].

**Fig. 2.**
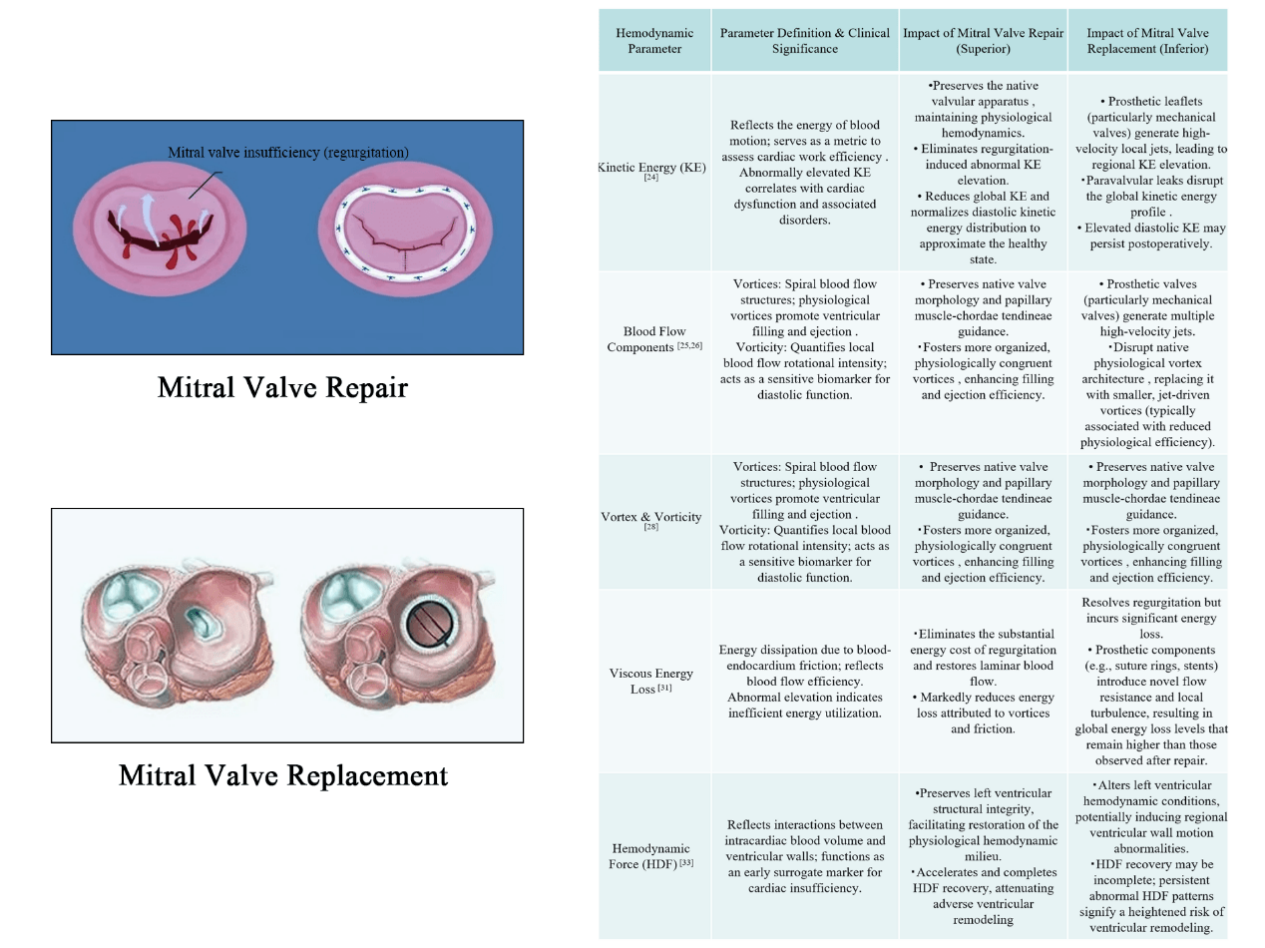
**Comparison of main hemodynamic parameters after mitral valve repair and replacement surgery**.

From the hemodynamic perspective of 4D Flow CMR, mitral valve repair is generally superior to replacement in restoring efficient, physiological intracardiac flow patterns. It better restores normal kinetic energy distribution, flow components, and vortex structure and minimizes energy loss, thereby providing greater benefits for cardiac function and long-term prognosis (Fig. 2, Ref. [24,25,26,28,31,33]). This offers strong hemodynamic support for clinically prioritizing repair. When replacement is necessary, 4D Flow CMR can meticulously assess the hemodynamic performance of different prosthetic valves and monitor subtle postoperative changes in cardiac function [37,38,39].

## 5. Inherent Limitations of Prosthetic Valves: Hemodynamics Post Mitral Valve Replacement

Prosthetic valves used in replacement are divided into mechanical and bioprosthetic valves: ① Patients receiving mechanical valve replacement require lifelong anticoagulation therapy, exposing them to higher risks of thromboembolic and hemorrhagic adverse events. A study following patients who underwent mitral mechanical valve replacement for rheumatic heart disease for up to 25 years showed a cumulative incidence of thromboembolic events of 11.0%, with rates of bleeding, non-structural valve dysfunction, and infective endocarditis increasing over time. Furthermore, as the condition progresses, patients with prior mitral mechanical valve replacement face correspondingly increased surgical risks for subsequent aortic valve replacement [1]; ② Bioprosthetic valves avoid the need for lifelong anticoagulation, with lower rates of anticoagulation-related bleeding and thromboembolic complications. However, their durability is limited, exhibiting higher reoperation rates and long-term mortality risks compared to mechanical valves [40,41].

To systematically evaluate the impact of mitral valve replacement on intra-left ventricular hemodynamics, a research team used an *in vitro* experimental platform to compare the performance of mechanical and bioprosthetic valves in the left ventricular flow field, focusing on vortex structure and differences in pressure distribution. The results showed that while mechanical valves maintained transvalvular pressure gradients similar to native valves, their bileaflet design formed three mutually interfering jets, causing reversal of the main left ventricular vortex direction and significantly increasing flow field complexity, especially with vertical valve implantation, in which vortex quantity distribution showed obvious asymmetry. Bioprosthetic valves could form a complete vortex ring during diastole aligned with the native valve direction, but their jet velocity was significantly higher than that of the native valve, and a high-pressure gradient persisted on the ventricular wall at end-diastole, potentially enhancing fluid forces on the ventricular wall. Native valves exhibited optimal hemodynamic efficiency: diastolic flow fully flushed the apical region, effectively reducing blood residence time; however, neither prosthetic valve type enabled vortex structures to fully extend into the apex, potentially increasing the risk of blood stasis and thrombosis in that region [42,43].

Whether mechanical or bioprosthetic, mitral valve replacement alters the intra-left ventricular blood flow structure, manifested as increased ventricular work and pumping load, altered ventricular wall stress patterns inducing adverse remodeling, prolonged blood cell residence time within the ventricle, thereby increasing thrombotic risk, and elevated flow field shear stress leading to potential hemolysis [44,45]. Overall, the significant advantages of valve repair over replacement lie not only in avoiding long-term anticoagulation and related complications, which improve valve durability and long-term prognosis, but more importantly, in maximally preserving the valve’s physiological structure and function [46]. Repair surgery, by reconstructing sufficient leaflet coaptation area and height, restores its normal closing morphology. This plays a vital role in maintaining left ventricular hemodynamic stability and preserving long-term cardiac function (Table 1).

**Table 1.** **Comparative table of hemodynamic decision-making for mitral valve surgery strategies**.

Decision dimension	Specific strategy / classification	Definition & key features	Core hemodynamic impact	Core advantages	Main limitations / challenges	Applicable scenarios / considerations
Overall strategy	Mitral Valve Repair	Preserves and reconstructs the native valve structure and function.	Restores physiological flow: Re-establishes a single, efficient large vortex with minimal energy loss and optimal flow organization.	1. Optimal hemodynamics 2. Avoids lifelong anticoagulation 3. Preserves ventricular function 4. Superior long-term survival	1. Highly dependent on surgeon expertise 2. Lower success rate for complex pathologies (e.g., rheumatic, severe calcification)	First choice for repairable anatomy (e.g., degenerative disease).
Replacement - valve type	Mechanical Valve	Manufactured from artificial materials, primarily bileaflet design, requires lifelong anticoagulation.	Non-physiological multi-jet flow: Creates central and lateral jets that split/disturb the physiological vortex. Higher energy loss.	1. Excellent durability 2. Effectively corrects regurgitation	1. Requires lifelong anticoagulation (bleeding/thrombosis risk) 2. Non-physiological hemodynamics	Suitable for younger patients, no anticoagulation contraindications, long life expectancy.
	Bioprosthetic Valve	Made from biological tissue, categorized into stented and stentless.	Diverse jet characteristics: Stentless valves approximate physiological vortex better; stented valves have flow obstruction. Generally superior to older mechanical valves.	1. Avoids lifelong anticoagulation 2. Stentless valves offer better hemodynamics	1. Risk of structural valve deterioration 2. Stented valves have slightly inferior hemodynamics	Suitable for older patients, those unwilling to undergo anticoagulation, life expectancy matching valve durability.
Replacement - design generation (subtype)	Contemporary Optimized Design (e.g., bileaflet mechanical, stentless bioprosthesis)	Fluid-dynamically optimized to reduce flow resistance and turbulence.	Reduced interference: More symmetric jets, larger effective orifice area, relatively lower energy loss.	Provides the best hemodynamic performance within its respective category.	Still cannot fully replicate the native valve’s flow-guiding function.	Should be prioritized when replacement is decided upon.
	Older Design (e.g., monoleaflet mechanical, early stented valves)	Early products with suboptimal fluid dynamic design.	Significant flow disruption: Produces high-turbulence, asymmetric jets, high energy loss, severely disrupts vortex.	Primarily of historical use; current evidence clearly indicates hemodynamic disadvantages.	Poor hemodynamic performance, a risk factor for adverse outcomes.	Largely superseded by contemporary designs; should be avoided in clinical practice.
Replacement - core cechnique	Preservation of Native Subvalvular Apparatus (Chordae & Papillary Muscles)	The patient’s own chordal system is not resected or is preserved during surgery.	Preserves ventricular foundation: Maintains ventricular geometry and contraction coordination, enabling relatively organized flow.	1. Protects left ventricular function 2. Reduces post-operative heart failure risk 3. Improves long-term prognosis	Technically demanding, not applicable to all anatomies.	A mandatory goal in replacement surgery; every technical effort should be made to achieve it.
	Resection of Native Subvalvular Apparatus	The patient’s chordae and papillary muscles are completely resected.	Impairs ventricular function: Leads to ventricular dilation, sphericity, fundamentally worsens flow, increases energy loss.	No clear advantage, primarily a historical technique or last resort for complex pathologies.	A major cause of post-operative left ventricular dysfunction and adverse remodeling.	Should be avoided whenever possible, considered only in exceptional circumstances.
Interventional therapy	Transcatheter Edge-to-Edge Repair (TEER)	Minimally invasive interventional technique using a clip to coapt the leaflets.	Dual-orifice, dual-jet flow: Creates two independent jets, disrupts overall vortex structure, increases energy loss.	1. Minimally invasive, no sternotomy 2. Suitable for surgically high-risk patients 3. Avoids cardiopulmonary bypass	1. Non-physiological hemodynamics 2. Potential for residual stenosis or regurgitation 3. Long-term durability data accumulating	A defined alternative for patients deemed high-risk/inoperable for surgery due to age, comorbidities, etc.

Both *in vitro* experiments and clinical studies have confirmed that whether it is a mechanical valve or a biological valve, replacement surgery will change the natural flow structure within the left ventricle, potentially leading to increased workload, abnormal wall shear stress, and prolonged blood retention time. Therefore, the choice of the surgical treatment is as important as the selection of the valve. The core advantage of valve repair surgery lies not only in avoiding complications related to artificial valves, but also in its ability to maximize the retention of the native valve structure and its ability to guide blood flow, fundamentally maintaining the physiological basis of left ventricular hemodynamics.

## 6. Reshaping Intracardiac “Vortices”: The Core Advantage of Mitral Valve Repair

Intra-left ventricular vortices play a key role in maintaining normal cardiac function, but their structure is susceptible to significant influence from mitral valve disease and surgical intervention. These hemodynamic changes may induce dysfunctional intracardiac vortices, thereby activating pathophysiological pathways associated with progressive left ventricular remodeling and thrombus formation.

**Fig. 3.**
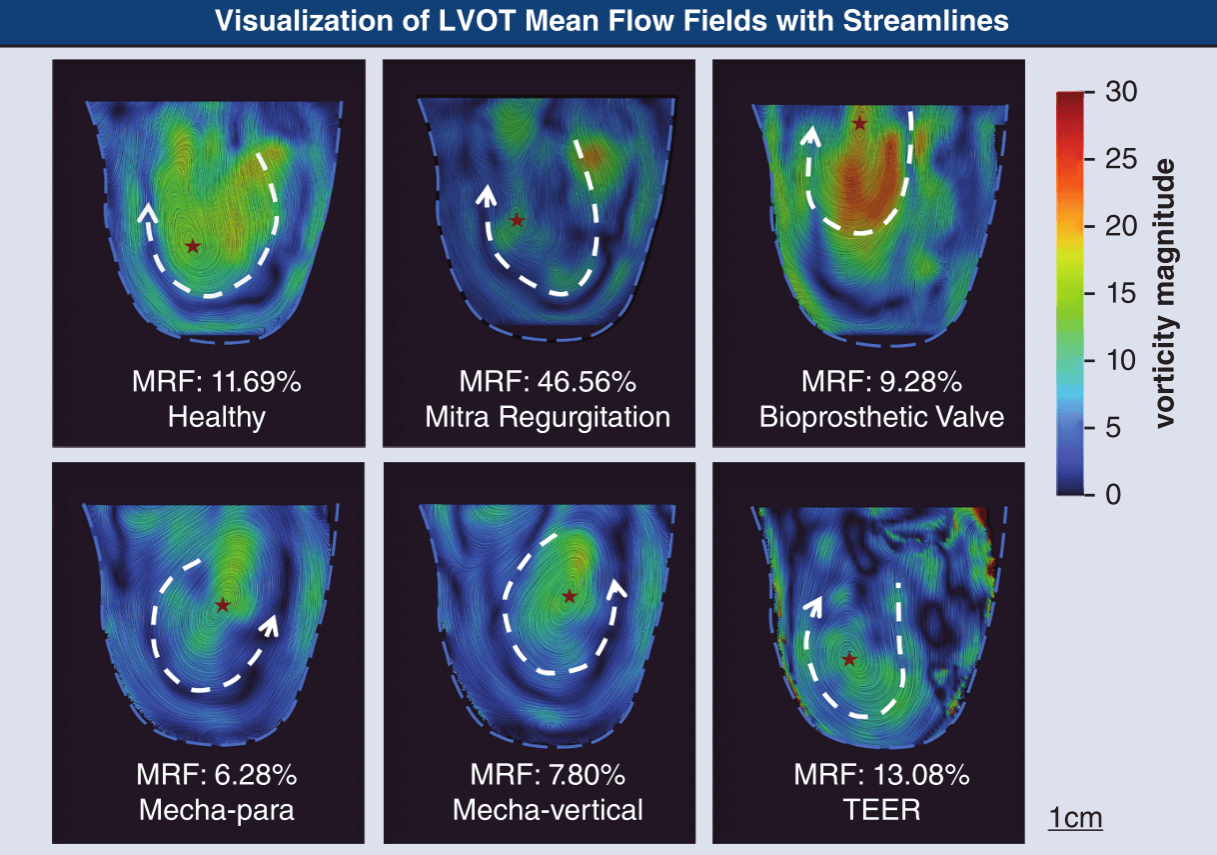
**Comparative analysis of left ventricular hemodynamic under different physiological and pathological conditions**. The study simulated six different valve states and presented the morphologies of the valve contraction and relaxation phases in each state. The flow direction is indicated by white arrows, and the size of the vortices is represented by colors. The center of the vortex is indicated by a red star. The lines in the figure represent distances, and the scale is marked as “1 centimeter” with annotations. Healthy,bioprosthetic valve; Mecha-para (parallel to the natural valve), mechanical valve; Mecha-vertical, mechanical valve (perpendicular to the natural valve); TEER, transcatheter edge-to-edge mitral valve repair; MRF, mitral valve fraction; LVOT, left ventricular outflow tract. Image from “*Impact of mitral valve interventions on left ventricular hemodynamics: Insights into energy loss and flow dynamics*” Reproduced with permission from Feng et al. [30].

To systematically assess this physiological mechanism, a research team employed a bio-hybrid *in vitro* platform to comparatively analyze left ventricular hemodynamics under physiological and pathological conditions. Using four-dimensional particle image velocimetry, they precisely measured multiple parameters of the left ventricular flow field, including mean velocity distribution, vortex depth and lateral position, viscous shear stress, and intensity of energy dissipation (Fig. 3, Ref. [30]). The findings revealed that isolated mitral regurgitation, while altering flow field characteristics to some extent, largely preserved the main vortex structure; however, different mitral valve surgical procedures had more profound and distinct impacts on left ventricular flow patterns: ① Mechanical valve replacement altered the original ventricular flow direction, reversing the originally clockwise main vortex to counterclockwise, causing flow deviation from the left ventricular outflow tract and reducing pumping efficiency [47]; ② Bioprosthetic valve replacement, while anatomically closest to the native valve, caused an overall upward shift of the vortex, increasing the risk of apical blood stasis. From a fluid dynamics perspective, this is more likely to induce thrombus formation [48]; ③ Transcatheter edge-to-edge repair (TEER), while effectively reducing regurgitation, formed dual-inlet jets due to the creation of a double orifice, disrupting the original coherent vortex structure, resulting in significantly increased energy loss and shear stress, indicating elevated cardiac energy expenditure and flow disorder [36,49]; ④ Mitral valve repair, by maximally preserving leaflets and subvalvular structures, maintained the conditions for generating physiological blood flow vortices, contributing to an efficient, low-energy-consumption pumping process and effectively avoiding potential hemodynamic disturbances post-replacement [50,51].

Preserving natural valve function optimizes left ventricular hemodynamics, whereas valve replacement and TEER both alter flow patterns, increasing cardiac load and the risk for thrombotic events. These findings emphasize the importance of assessing left ventricular hemodynamics in the treatment of mitral regurgitation. The impact of different procedures extends far beyond altering valve orifice morphology or closing mechanism; they reshape the fluid dynamic environment within the left ventricle at a deeper level [30]. The mitral valve is not an isolated “valve” but a key hub within the cardiac dynamic system. Therefore, the ultimate goal of surgery should not be limited to eliminating regurgitation or enlarging the orifice, but also to maintaining or reconstructing the hemodynamic “order” within the left ventricle.

However, in the postoperative cardiac surgical assessment, a series of imaging parameters are often used as criteria for judging surgical success, such as postoperative disappearance of regurgitation, reduced transvalvular pressure gradient, and adequate valve orifice area [52,53]. These data often appear satisfactory on the surface. Yet, during clinical follow-up, a puzzling phenomenon gradually emerges: some patients, despite significant improvement in echocardiographic indicators and near-elimination of regurgitation, do not recover cardiac function as expected, and still exhibit reduced exercise tolerance, low cardiac function indices, and suboptimal long-term prognosis. Against this background, the surgical choice for treating mitral valve disease-repair vs. replacement-may become a key factor influencing functional prognosis. Focusing on fluid dynamics in valve surgery may provide us with new theoretical foundations [54].

The mitral valve, compared to prosthetic valves, plays an irreplaceable role in maintaining left ventricular structural and functional integrity, primarily manifested in two aspects: ① Optimizing diastolic filling and enhancing myocardial contraction efficacy: During diastole, the intact mitral apparatus, through its tethering effect on the left ventricle, promotes more complete transverse ventricular filling, thereby achieving ideal preload. The greater the number of functional units (cross-bridges) participating in contraction within the myocardium, the stronger the contractile force. Preserving the mitral valve and its subvalvular structures maintains coordinated longitudinal tension in the myocardium, ensuring more muscle fibers are in an optimal preparatory state before contraction. If valve replacement is performed, especially with excision of subvalvular structures, this tethering effect is lost (i.e., longitudinal filling), leading to a significant reduction in myocardial units (cross-bridges) participating in effective contraction during the ejection phase. Therefore, the heart must compensate by increasing work to maintain output, imposing a long-term burden on the heart. ② Guiding sequential contraction and enhancing hemodynamic efficiency: Myocardial contraction is not a simple “all-or-none” mode but a coordinated, spiral sequence of activity from apex to base. The mitral apparatus plays a crucial role in tension transmission in this process, prompting the formation of a functionally significant “large vortex” within the left ventricle before ejection. This blood flow vortex itself possesses kinetic energy reserve, significantly reducing the external work required for cardiac ejection, lightening the overall load, and thereby improving pumping efficiency [30,55].

In summary, cardiac ejection relies on the combined action of effective myocardial contraction and the intrinsic kinetic energy of blood flow, both working together to overcome systemic vascular resistance. Weakening of either function will force the myocardium into a compensatory overload state. Long-term development inevitably leads to decompensation, ultimately causing continuous deterioration of cardiac function. Therefore, in mitral valve surgery, maximal preservation of its structural and functional integrity is crucial for maintaining long-term left ventricular function. In addition, the endocardial surface is not smooth; it consists of papillary muscles, variously shaped trabeculae carneae, and chordae tendineae, collectively forming a complex three-dimensional space that significantly influences intraventricular flow patterns. Studies using left ventricular endocardial anatomical models have found that the presence of trabeculae and papillary muscles increases energy loss within the ventricle while reducing ventricular wall shear stress. When mitral valve structural integrity is compromised, multiple secondary small vortices are generated in the flow, significantly weakening the original vortex kinetic energy of the blood [56,57,58]. This indicates that the inherent physiological structure of the endocardium, by altering the distribution and dissipation of flow energy, directly affects the hemodynamic state within the cardiac chambers, an indispensable component for maintaining efficient cardiac pumping function (Table 2, Ref. [59,60,61,62]).

**Table 2.** **Multi-dimensional comparison: mitral valve repair vs. replacement [59,60,61,62]**.

	Mitral valve repair	Mitral valve replacement
Long-term survival	Significant and sustained advantage in patients with degenerative disease and infective endocarditis.	Generally lower than repair, but a necessary choice in certain situations.
Reoperation risk	Lower reoperation rate in degenerative disease.	Reoperation risk varies by etiology, e.g., valve-in-valve procedures.
Cardiac function recovery	Clear advantage, better preservation of left ventricular structure and function, superior postoperative improvement in cardiac function indices.	It may damage the continuity of the left ventricle-annulus, and has an adverse effect on the long-term maintenance of cardiac function.
Hemodynamics	Maintains more physiological hemodynamic state; postoperative improvement in indices like left ventricular and atrial size is more significant.	Alters original physiological structure; degree of hemodynamic improvement is typically inferior to repair.
Main advantages	Clear survival benefit; no lifelong anticoagulation (primarily vs. mechanical replacement); maintains normal cardiac physiological structure and function.	Applicable for complex lesions unsuitable for repair; provides definitive correction of valve function.
Main limitations	High demands on surgeon skill and experience; in ischemic and rheumatic disease, long-term survival advantage may not be clear and reoperation rate may be higher.	Mechanical valves require lifelong anticoagulation, increased bleeding and thrombotic risks; bioprosthetic valves have durability issues. Both may impair LV function.

LV, left ventricle.

Both *in vitro* experiments and clinical studies have confirmed that regardless of whether it is a mechanical valve or a biological valve, valve replacement surgery will change the natural flow structure within the left ventricle, potentially leading to increased working load, abnormal wall shear stress, and prolonged blood flow retention time, among other issues. Therefore, the choice of the surgical approach is as important as the selection of the valve in terms of its impact on hemodynamics. The core advantage of valve repair surgery lies not only in avoiding complications related to artificial valves but also in fundamentally maintaining the physiological basis of left ventricular hemodynamics by preserving the native valve apparatus and its ability to guide blood flow to the greatest extent possible.

## 7. Current Status, Challenges, and Future Directions of Interventional Mitral Valve Repair

In the future field of cardiovascular research, with the widespread dissemination of minimally invasive and interventional concepts, using interventional techniques and simulated leaflet devices to enhance anterior-posterior leaflet coaptation, correct leaflet prolapse, and address insufficient coaptation area causing regurgitation is becoming a promising therapeutic direction. Such techniques not only effectively improve regurgitation but also preserve the physiological single-orifice structure of the mitral valve during diastole, thereby avoiding the risk of mitral stenosis.

Currently, only transcatheter edge-to-edge repair (TEER) is a relatively proven technique among interventional mitral repair therapies, having been applied in over hundreds of thousands of cases worldwide and recommended by domestic and international guidelines [61,63]. Therefore, we must recognize that TEER holds significant value in specific patient populations. For example, in patients deemed high surgical risk or ineligible due to advanced age, severe comorbidities, severely impaired left ventricular function, or a history of prior cardiac surgery, TEER offers a minimally invasive solution with low trauma and no need for cardiopulmonary bypass. Large randomized controlled trials (e.g., the cardiovascular outcomes assessment of the MitraClip percutaneous therapy for heart failure patients with functional mitral regurgitation (COAPT trial)) have confirmed that for patients with severe secondary mitral regurgitation refractory to medical therapy, TEER significantly reduces all-cause mortality and rates of heart failure rehospitalization, effectively improving symptoms and quality of life [36,64]. In this large high-risk patient group, the advantages of TEER (accessibility, safety, symptom improvement) far outweigh its non-physiological hydrodynamic defects, making it an important “bridge” between medication and surgery. However, it still has some apparent shortcomings: post-TEER, the mitral orifice changes from single to double, which may lead to mitral stenosis; mitral jets transform into two independent jets diverging from the valve, impacting the opposite ventricular wall, generating higher turbulence, altered shear stress, and intraventricular pressure gradients. Furthermore, the impact of high-velocity blood flow may cause clip deformation, leading to changes in valve tissue structure, or make re-intervention post-TEER difficult (Table 3) [65,66,67,68,69].

**Table 3.** **Comparison of clinical application and considerations of different mitral valve intervention strategies**.

	**Repair**	**Replacement**	**TEER**	**Secondary MR patients at high surgical risk/contraindicated (COAPT-like population)**
Core therapeutic goal	Anatomical and functional dual reconstruction, pursuing optimal hemodynamics.	Safe and effective correction of regurgitation.	Effective correction of regurgitation while maximizing left ventricular function preservation by retaining chordae.	Reduce mortality and heart failure hospitalization risks, improve quality of life.
Hemodynamic outcome	Theoretically optimal. Most likely to restore physiological vortices, low energy loss, and efficient kinetic energy distribution.	Highly variable. Outcome heavily depends on surgeon’s skill; ideal hemodynamic reconstruction may not be achieved.	Superior to traditional replacement (with chordal resection). Better maintains ventricular geometry and contractile coordination, but the prosthetic valve still introduces non-physiological jets and vortices.	Non-physiological. Creates a double-orifice, dual-jet flow, disrupting the overall ventricular vortex structure and increasing energy loss. However, its clinical benefits have been confirmed in specific populations, where its hemodynamic “disadvantages” are outweighed by its accessibility and safety advantages.
Thrombotic/Bleeding risk	Lowest.	Mechanical valves require lifelong anticoagulation.	Mechanical valves require lifelong anticoagulation. Bioprosthetic valves require short-term anticoagulation (usually 3–6 months) post-op, with low long-term risk.	Low. Typically requires only dual antiplatelet therapy, no long-term potent anticoagulation.
Long-term clinical outcome evidence	In degenerative disease, significant advantages over replacement in survival, reoperation rates, and cardiac function preservation have been proven.	Evidence is less abundant, and outcomes are often inferior to those at expert centers.	Chordal preservation is the standard technique. Compared to replacement with chordal resection, it improves postoperative cardiac function and survival, but still generally inferior to successful repair.	In the population with “severe secondary MR refractory to medical therapy and at high surgical risk”, large RCTs (COAPT) have confirmed significant reductions in mortality and heart failure hospitalization rates.
Main limitations/challenges	1. Highly dependent on surgeon’s experience and center expertise. 2. Limited success rate and durability for complex pathologies (e.g., rheumatic, ischemic, severe calcification).	Risk of repair failure requiring conversion to replacement may be higher.	1. Inherent limitations: complications related to prosthetic valves (structural deterioration, thrombosis, hemolysis, etc.). 2. Hemodynamic efficiency always lower than ideal native valves.	1. Strict patient selection (anatomical suitability). 2. Potential for residual/recurrent regurgitation, mitral stenosis. 3. Long-term durability data is still accumulating.

MR, mitral regurgitation; RCT, randomized controlled trial; COAPT, cardiovascular outcomes assessment of the MitraClip percutaneous therapy for heart failure patients with functional mitral regurgitation.

Compared to transcatheter aortic valve replacement (TAVR), the clinical application and dissemination of transcatheter mitral valve repair are relatively lower, mainly due to unique challenges posed by mitral valve anatomy: the surrounding area lacks vascular structures similar to the aortic valve region for stable stent anchoring. Thus, achieving stable anchoring of the prosthesis in the dynamic, beating mitral annulus plane becomes a key technical challenge [70,71]. Additionally, the emergence of Partial Heart Transplantation (PHT, also known as cardiac valve transplantation) brings new hope to this therapeutic dilemma. A 2024 JAMA report described surgeons transplanting only the aortic or pulmonary root and valve tissue from a donor heart, while preserving the recipient’s own ventricles. This “living valve” contains intact cellular structures, capable of continued metabolism, repair, and growth within the recipient’s body. PHT is not only a new surgical technique but a key turning point for cardiac surgery, translating theory into practice, making “growable valves” a reality, and offering unprecedented therapeutic possibilities for complex valvular disease [72,73].

Therefore, in treating MR, preserving natural valve function is crucial for optimizing left ventricular hemodynamics. Numerous studies have found that mechanical valves and TEER may increase cardiac load, while bioprosthetic valves may elevate the risk of left ventricular apical thrombus formation. These results suggest that in choosing valve intervention strategies, we should emphasize how to maximally reduce energy loss, minimize thrombotic risk, and suppress adverse cardiac remodeling.

## 8. Discussion and Future Perspectives: From Hemodynamic Understanding to Clinical Practice Revolution

Valve surgery has entered an era of precision centered on “long-term patient prognosis”. However, it must first be clearly understood that advocating “repair first” based on hemodynamic advantages does not mean that repair surgery is the absolutely preferred option in all situations. The value of hemodynamic thinking lies in providing a crucial decision-making dimension and optimization goal for the selection of individualized surgical strategies, rather than a simple either-or judgment. This review systematically demonstrates the core advantages of mitral valve repair in maintaining physiological left ventricular hemodynamics, surpassing traditional imaging targets such as “valve orifice area, regurgitation severity, transvalvular pressure gradient”. It reveals that re-establishing intracardiac “flow field order” is key to achieving efficient, low-energy-consumption cardiac pumping and favorable long-term function. This hemodynamic perspective provides a solid scientific foundation for the “repair-first” principle and guides the future direction of valve surgery [74].

To translate this understanding into clinical practice, we must bridge the gap from “theoretical knowledge” to “routine practice”. Future efforts should focus on the following three levels, constructing a complete translational pathway:

### 8.1 Clinical Decision Transformation: Integrating Hemodynamic Indicators Into Individualized Treatment Plans

Current clinical decisions are largely based on etiology, anatomical feasibility, and traditional echocardiographic parameters. In the future, hemodynamic assessment should become an important component of the decision matrix.

Preoperative Navigation: Utilize 4D Flow CMR or computational fluid dynamics simulations based on routine imaging to perform a “functional prediction” of the patient’s left ventricular flow field. For example, for patients with secondary MR and significantly dilated left ventricles, if assessment shows their physiological vortex structure is completely disrupted, simple repair may be insufficient to reconstruct an efficient flow field. In such cases, a comprehensive evaluation of the hemodynamic consequences of combined strategies such as repair, replacement, or even ventricular remodeling is needed.

Intraoperative Guidance and Postoperative Prognostic Judgment: Parameters like vortex kinetic energy and energy loss can serve as real-time “biosensors” of surgical outcomes. Future development of simpler intraoperative flow field monitoring techniques (e.g., transesophageal echocardiography-derived vortex assessment) could help surgeons immediately judge the quality of repair. Postoperatively, these parameters can become sensitive markers for predicting functional recovery and long-term prognosis, far superior to delayed changes in ventricular dimensions.

### 8.2 Future Research Directions: From Mechanistic Exploration to Technological Empowerment

Establishing Standardized “Hemodynamic Biomarkers”: Through large-scale prospective cohort studies, define physiological ranges and pathological thresholds for key hemodynamic parameters (e.g., apical vortex strength, diastolic direct flow ratio, global viscous energy loss) and correlate them with clinical hard endpoints (e.g., heart failure rehospitalization, mortality), establishing them as recognized assessment standards.

Driving “Hemodynamically-Optimized” Precision Surgical Techniques: Hemodynamics should become the theoretical guide for optimizing surgical techniques. Research the impact of different annuloplasty ring types and sizes on vortex morphology; explore optimal number, location, and tension for artificial chordae implantation to achieve precise regulation of flow guidance. Personalized computational fluid dynamics (CFD) simulation platforms based on patient-specific imaging could allow virtual testing of different repair strategies preoperatively, realizing a paradigm shift of “inferring the optimal procedure from hemodynamic outcomes” [75].

Guiding Iteration and Innovation of Interventional Devices: Addressing the energy loss issues caused by dual-orifice jets post-TEER, research should focus on how to minimize hydrodynamic drawbacks by optimizing clip design (e.g., streamlined profile) and implantation strategy (target location and grasp extent). This also increases demand for next-generation interventional devices-those that not only reduce regurgitation but also actively restore or mimic physiological vortices.

### 8.3 Clinical Translation Pathway: Promoting Technology Dissemination and Conceptual Innovation

Technological Simplification and Automation: Promote automation and standardization of post-processing workflows for technologies like 4D Flow CMR. Develop “one-click” hemodynamic analysis modules integrated into clinical workstations, transforming them from advanced research tools into “routine equipment” readily accessible to clinicians.

Guideline Integration and Educational Reform: When sufficient evidence accumulates, incorporate core hemodynamic assessment recommendations into domestic and international valvular heart disease management guidelines and consensus statements. More importantly, integrate basic knowledge of cardiac fluid dynamics and image interpretation into the training systems for cardiac surgery and cardiology specialists, fundamentally cultivating a generation of clinicians with “flow-field thinking”.

### 8.4 Insights Into Mechanisms and Clinical Evidence: The Current Gap

Current research in the field of fluid mechanics in mitral valve surgery presents a significant gap between “in-depth mechanism insights” and “lagging clinical evidence”:

Mechanistic studies are extensive, but the overall quality of the evidence remains limited. Existing studies, through techniques such as *in vitro* simulation, CFD, and 4D Flow CMR imaging, clearly explain the significant advantages of mitral valve repair in maintaining physiological vortices, reducing energy loss, and optimizing kinetic energy distribution from a biophysical perspective. These findings provide solid theoretical rationale and mechanism explanations for the “repair priority” principle.

The lack of direct clinical evidence connecting fluid mechanics with hard endpoints: Although the mechanism research strongly suggests that abnormal flow patterns (such as vortex disorder, increased energy loss) will lead to adverse ventricular remodeling, inefficiency, and increased risk of thrombosis, which will affect long-term prognosis (death, re-hospitalization for heart failure), this causal chain has not yet been directly confirmed in large-scale prospective clinical studies. Most of the current evidence comes from *in vitro* experiments, small sample observational studies, or retrospective analyses.

Key gap: The prognostic value of fluid mechanics parameters has not been established. Currently, there is no high-level evidence indicating that specific fluid mechanics parameters (such as vortex intensity, direct flow ratio, global viscous energy loss) can be used as independent biomarkers for hard clinical endpoints (such as all-cause mortality, stroke, re-hospitalization for heart failure). Whether it can surpass and complement traditional clinical and ultrasound parameters still needs to be verified by large-scale, prospective cohort studies.

Challenges in Technology Transfer and Standardization: The routine application of complex flow field assessment techniques, such as 4D Flow CMR, in clinical decision-making still faces practical barriers, such as complex analysis processes, time-consuming nature, and the lack of unified reference values.

Although the fluid mechanics perspective provides a profound and convincing new paradigm for understanding the differences in mitral valve surgery, the leap from “mechanism correlation” to “clinical causality” has not yet been completed. Future research urgently needs to bridge this gap by conducting rigorous prospective clinical studies to verify the independent predictive value of fluid mechanics indicators for patients’ long-term outcomes, thus truly achieving the transformation from theoretical cognition to evidence-based practice.

### 8.5 Pathophysiological Mechanisms of Disordered Flow Fields Leading to Adverse Remodeling and Thrombosis Risk

#### 8.5.1 Core Pathways Leading to Adverse Left Ventricular Remodeling:

① Abnormal Signal Initiation: Abnormal wall shear forces cause endothelial dysfunction in the endocardium, activating pro-fibrotic (transforming growth factor-β) and pro-hypertrophic (Endothelin-1 (ET-1)/mitogen-activated protein kinase (MAPK)) signaling pathways.

② Energy Metabolism Imbalance: Ineffective energy loss forces myocardial metabolic remodeling (shift to inefficient glycolysis) and damages mitochondrial function, leading to energy depletion and decreased contractility over the long term.

③ Mechanical Stress Changes: Local blood stasis and increased abnormal pressure gradients cause uneven stress on the ventricular wall, directly stimulating myocardial hypertrophy and damaging microcirculation.

#### 8.5.2 Key Steps Increasing Thrombosis Risk:

① Creating a Stagnation Environment: Stagnation of blood flow at the apex leads to prolonged local contact and activation of coagulation factors.

② Direct Damage to Cells: High shear forces and turbulence cause red blood cell rupture (release of adenosine diphosphate and other pro-coagulant substances) and abnormal activation of platelets.

③ Altering Vascular Internal Balance: Abnormal flow fields cause the endothelial phenotype to shift from anti-coagulant (nitric oxide, thrombomodulin) to pro-coagulant (release of von Willebrand factor, tissue factor expression), and inhibit fibrinolysis.

Disordered left ventricular flow fields are not merely a “low efficiency” flow mechanics phenomenon; they directly act on endothelial cells, myocardial cells, and blood components, activating a cascade reaction from molecules to cells to tissues. These reactions are interwoven and mutually reinforcing, jointly forming a clear pathological physiological pathway that connects the two key clinical endpoints of “abnormal blood flow” and “adverse ventricular remodeling/thrombosis”. This provides a profound mechanistic explanation as to why restoring the physiological “flow field order” is crucial for improving the long-term prognosis of patients.

## 9. Limitation

This study suggests that some hemodynamic parameters after mitral valve surgery may have an adverse effect on the long-term recovery of cardiac function. This viewpoint is reasonable based on biological and fluid dynamics principles, but the causal relationship in the human body has not yet been clearly confirmed. Currently, most supporting data come from in vitro experiments, CFD simulations, or small-scale observational imaging studies. If this finding can be incorporated into clinical research design and efficacy evaluation in the future, it may further validate its clinical significance.

## 10. Conclusions

We should not simplistically claim that mitral valve repair is superior to replacement in all circumstances (i.e., a poor repair is worse than prosthetic valve replacement). The choice of specific procedure must be based on a comprehensive assessment of the patient’s actual condition (e.g., clarity of regurgitation/stenosis etiology, physical ability to tolerate surgery, integrity of leaflets and subvalvular structures, etc). For example, in complex pathologies like rheumatic, ischemic, or severe calcific disease, valve repair is technically challenging with low success rates and high long-term reoperation rates; replacement remains a necessary choice in such cases [76]. However, for most clinical scenarios, current evidence-based medical data consistently indicate that compared to replacement, patients undergoing repair not only effectively reduce postoperative risks of complications such as thrombosis and infective endocarditis but also show significantly decreased rates of reoperation and increased long-term survival [77,78].

The ultimate goal of valve surgery is evolving from traditional “anatomical correction” towards the higher dimension of “hemodynamic functional reconstruction”. The 2025 European Society of Cardiology (ESC)/European Association for Cardio-Thoracic Surgery (EACTS) Guidelines for the management of valvular heart disease state: assessment of valvular disease should not only focus on the valve itself but also its impact on left ventricular, left atrial, and right ventricular function, and pulmonary artery pressure, as this directly relates to prognosis and timing of interventions [79].

**Fig. 4.**
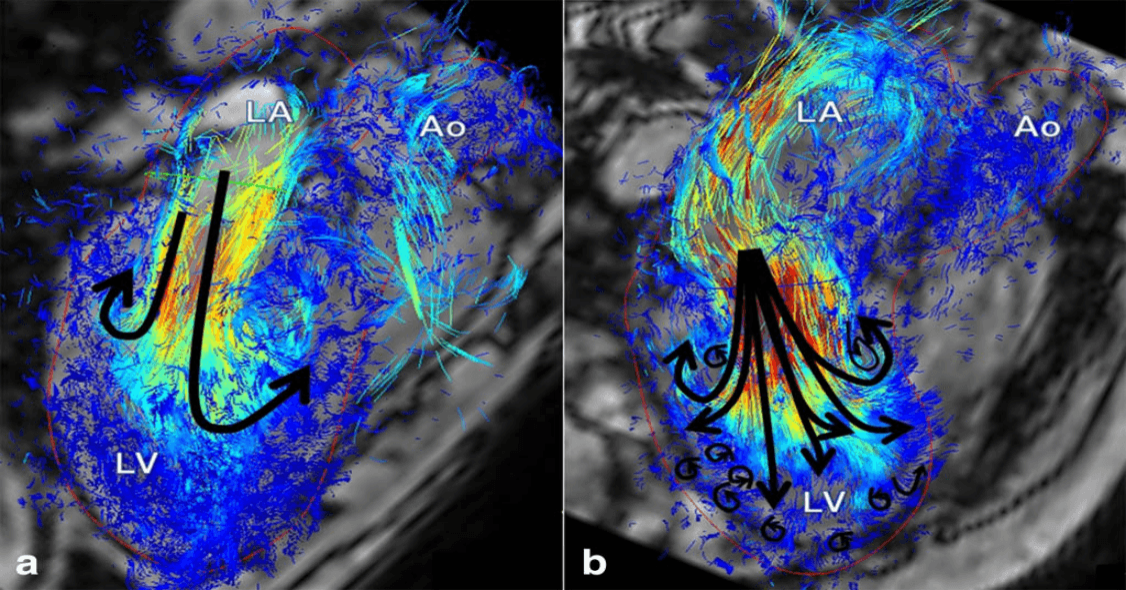
**Comparison of the distribution of left ventricular diastolic blood flow during healthy conditions and mitral valve regurgitation**. These images were obtained through 4D flow MRI using particle tracking. In the healthy left ventricle (LV) (a), the main part of the blood flow follows a smooth path from the left atrium to the aorta, while below the mitral valve there is a reverse-rotating vortex. In contrast, in the case of mitral valve regurgitation (b), a larger proportion of the circulating blood flows outward from the left ventricular outflow tract and several vortices are scattered around the left ventricle. The black arrows indicate the path and direction of the main flow part and the flow direction of the vortex part. LA, left atrium; LV, left ventricle; Ao, Aorta. (Reproduced with permission from Al-Wakeel N, Fernandes JF, Amiri A, Siniawski H, Goubergrits L, Berger F, et al; published by Journal of Magnetic Resonance Imaging : JMRI. 2015; 42: 1705–1712. Image from “*Hemodynamic and energetic aspects of the left ventricle in patients with mitral regurgitation before and after mitral valve surgery*” [80]).

Furthermore, a study evaluated the KE characteristics of blood flow in the left ventricle of MR patients and analyzed the recovery effect of mitral valve surgery on the hemodynamic status [80]. Ten MR patients (before and after surgery for comparison) and seven healthy volunteers were examined using 4D blood flow magnetic resonance imaging (MRI) to obtain three-dimensional blood flow velocity data and calculate the average KE, peak KE during systole, and peak KE during early and late diastole. The conclusion states: ① Volume changes: The left ventricular volume of the patients before surgery was significantly higher than that of the healthy group, and it significantly decreased after surgery; ② Kinetic energy changes: The average KE and peak KE during early diastole before surgery were significantly increased, while the average KE, peak KE during systole and early diastole after surgery significantly decreased and approached normal, and the peak KE during late diastole after surgery remained persistently high and did not return to normal; ③ Blood flow patterns: The blood flow of the patients showed abnormal vortices and recirculation, and the improvement was partial but not complete. The hemodynamics of the MR patients were significantly abnormal. Surgery can partially improve it, but the kinetic energy during late diastole remained high and did not return to normal, suggesting that the physiological blood flow has not fully recovered. This study compared the hemodynamic parameters of the left ventricle before and after surgery to provide guidance for the recovery of long-term cardiac function and the selection of surgical methods (Fig. 4, Ref. [80]).

Therefore, integrating hemodynamic assessment into clinical decision-making does not replace traditional methods but provides a crucial new dimension, enabling our treatments to not only save lives but also enhance their quality. This pathway, though filled with challenges, is undoubtedly the necessary route towards a more precise, individualized, and prognostically superior future for valve surgery.
