# Supplementary material for: Mitral Valve Repair vs. Replacement: Hemodynamics in Valve Surgery
Source: Rev Cardiovasc Med. 2026 Jun 28;27(6):49299. doi: 10.31083/RCM49299 (PMC13339195; doi:10.31083/RCM49299)
Supplement: Supplementary file 1 [file 2153-8174-27-6-49299-s1.zip › supplementary material.doc]

1. MRI Scanner (Hardware)

Device: GE Discovery MR750w 3.0T system

Manufacturer: GE Healthcare

Location: Milwaukee, WI, USA

2. 4D Flow Post‑processing Software (Commercial)

Software: CAAS MR

Version:

version 5.1 used for primary analysis

version 5.2 (prototype) used for peak velocity assessment

Manufacturer: Pie Medical Imaging

Location: Maastricht, The Netherlands

3. 4D Flow Acquisition Sequence (Proprietary Technique)

Sequence Name: Kat‑ARC (also called HyperKat), a GE‑specific accelerated sequence; it is not a standalone software product and has no separate version/lot number.

Manufacturer: GE Healthcare (the sequence is embedded in the scanner software)

Location: Same as above (Milwaukee, WI, USA)

4. Statistical Software

Software: MedCalc Statistical Software

Version: version 20.011

Manufacturer: MedCalc Software Ltd.

Location: Ostend, Belgium

5.CFD solver

Version:lifex-cfd

Manufacturer:Open-source software

Location:SISSA, Trieste, Italy

6.In vitro left ventricular model

Version:3D-printed silicone left ventricular model

Manufacturer:Self-made by research team

Location:Beijing, China

7.PIV measurement system

Version:Tomographic Particle Image Velocimetry (Tomo PIV)-24

Manufacturer:TSI/LaVision

Location:Shoreview, Minnesota, USA/Göttingen, Germany

8.MRI scanner

Version:Specific version not specified

Manufacturer: Siemens

Location:Siemens Erlangen, Germany

**The "in vitro experimental platform" and "four-dimensional particle image velocimetry" system mentioned in the manuscript were mostly built by the research team themselves, and their specific software and hardware configurations varied depending on the research. To ensure the accuracy and reliability of the information, we reviewed relevant literature and found that the version numbers and manufacturer information recorded for the same equipment in different studies were inconsistent. Therefore, it is recommended that the above content be presented as supplementary materials separately, rather than included in the main text, to avoid misleading readers.**
